# Health-related quality of life and its determinants in children with allergic rhinitis

**DOI:** 10.3389/fped.2026.1817538

**Published:** 2026-06-03

**Authors:** Lei Guo, Guangzhen Lu, Xiaoting Wang, Ye Li

**Affiliations:** 1 Department of Otolaryngology-Head and Neck Surgery, Shandong Provincial Hospital Affiliated to Shandong First Medical University, Jinan, Shandong, China; 2 Department of Cardiology, Shandong Provincial Hospital Affiliated to Shandong First Medical University, Jinan, Shandong, China

**Keywords:** allergic rhinitis, children, health-related quality of life, PRQLQ, visual analog scale

## Abstract

**Background:**

Allergic rhinitis (AR) is highly prevalent in school-aged children and can substantially impair health-related quality of life (HRQoL). The modifiable determinants of HRQoL in this population remain incompletely understood.

**Methods:**

In this cross-sectional study, 110 children aged 6–12 years with physician-diagnosed AR and 110 age- and sex-matched healthy controls were enrolled from a tertiary hospital (January–December 2023). HRQoL was assessed using the Pediatric Rhinoconjunctivitis Quality of Life Questionnaire (PRQLQ). A visual analog scale (VAS) quantified four core nose symptoms, while a structured questionnaire captured household environmental exposures, allergen patterns, and treatment adherence. Anxiety and sleep quality were evaluated using HADS-A and PSQI, respectively. Independent-samples t tests, repeated-measures ANOVA with Bonferroni correction, and multiple linear regression were performed.

**Results:**

The Chinese PRQLQ demonstrated good reliability, with an overall Cronbach's *α* of 0.891 and an overall test–retest correlation coefficient of 0.895. Children with AR had higher total and domain PRQLQ scores than controls (all *P* < 0.01). Core nose symptoms were comparably severe, while secondary symptoms were milder. Poor HRQoL was independently associated with pet ownership, secondhand smoke, high indoor humidity, frequent pollen-season outdoor activity, increased dust-mite exposure, poor medication adherence, absence of allergen immunotherapy, higher anxiety, and poor sleep (all *P* < 0.01).

**Conclusions:**

AR markedly impairs HRQoL in school-aged children. Targeted management of nose symptoms, household exposures, adherence, and psychosocial factors, combined with timely allergen immunotherapy, may optimize quality of life.

## Introduction

Allergic rhinitis (AR) is a chronic, immunoglobulin E (IgE)-mediated inflammatory disease of the nasal mucosa driven by an abnormally activated T helper 2 (Th2) immune response (1). When sensitized children encounter airborne allergens such as pollen, house dust mites, or pet dander, large amounts of inflammatory mediators—including histamine and leukotrienes—are released, leading to the typical constellation of nasal and eye symptoms. The global prevalence of AR continues to rise; recent epidemiologic estimates suggest that between 500 million and 1.56 billion individuals are currently affected worldwide (2). In China, approximately 240 million people live with AR, and the clinical diagnosis rate among children aged 6–12 years is close to 15%, meaning that roughly one to two out of every ten school-aged children are affected (2). AR has now become the second most common health problem in student populations, surpassed only by myopia, underscoring the close link between environmental exposures and the development and progression of this condition.

Pediatric AR has a distinctive clinical profile. Studies have shown that persistent nasal obstruction (reported in >90% of cases), paroxysmal sneezing, and ocular itching are among the most common symptoms in children (3, 4). These pathophysiologic changes not only impair nasal airflow but also engage downstream neuro-immune pathways, such as histamine-H1 receptor signaling, which can set the stage for multisystem complications. Evidence from clinical and epidemiologic studies indicates that children with AR have approximately a 1.75-fold higher risk of moderate-to-severe obstructive sleep apnea (OSA) than their healthy peers [95% confidence interval (CI): 1.32–2.31], an attention-deficit/hyperactivity disorder (ADHD) detection rate of 26.4% (95% CI: 22.7–30.1), and anxiety and depression scores that are elevated by 1.5-to 3-fold (*P* < 0.001) (5, 6). In a cohort of British adolescents, seasonal AR symptoms occurring during examinations were associated with a 43% higher risk of poorer academic performance compared with unaffected students. Together, these findings highlight the wide-ranging impact of AR on children's physical health, emotional well-being, and school performance.

Assessing health-related quality of life (HRQoL) is therefore essential for capturing the true burden of AR in children. The Pediatric Rhinoconjunctivitis Quality of Life Questionnaire (PRQLQ) (7) covers five core dimensions—nose symptoms, eye symptoms, practical problems, other symptoms, and activity limitation—and has shown good cross-cultural adaptability and feasibility in clinical practice (Chinese version Cronbach's α=0.89, 95% CI: 0.85–0.92) (8). It is widely recommended as a standardized tool for evaluating HRQoL in children with AR. However, most studies in China have focused mainly on symptom profiles, with relatively little systematic work on modifiable factors such as environmental exposures [for example, fine particulate matter (PM_2.5_) concentrations and indoor allergen load], treatment adherence, and family management behaviors. This gap has limited the development of individualized, precision-based intervention strategies. Against this background, the present study aimed to use the PRQLQ to quantify the degree of HRQoL impairment in children aged 6–12 years with AR, and—by integrating data from environmental exposure questionnaires and treatment logs—to identify key modifiable determinants of HRQoL. Our goal was to provide a solid empirical basis for designing a “symptom-environment-behavior” tri-dimensional intervention model for pediatric AR.

## Methods

### Study population

This cross-sectional study consecutively enrolled eligible children from the outpatient Department of Otorhinolaryngology at our hospital between January and December 2023 in order to reduce potential selection bias. A total of 110 children aged 6–12 years with a confirmed diagnosis of allergic rhinitis (AR) were included as the observation group. During the same period, 110 healthy children who underwent routine health examinations and had no nasal disease were recruited as the control group.

The diagnosis of AR was based on the Chinese Guidelines for the Diagnosis and Treatment of Allergic Rhinitis (2022 revision) and required all of the following: (1) presence of ≥2 nasal symptoms (nasal congestion, rhinorrhea, sneezing, nasal itching) persisting or accumulating for ≥1 h per day; (2) pale, edematous nasal mucosa with watery secretions on physical examination; and (3) positive skin prick test or serum specific IgE to relevant allergens.

Inclusion criteria were: age 6–12 years; for the observation group, no systemic antihistamines or corticosteroids in the 4 weeks prior to enrollment; and signed written informed consent from a parent or legal guardian.

Exclusion criteria were: coexisting nasal polyps, chronic rhinosinusitis, or other nasal diseases; severe systemic disease involving the heart, liver, kidneys, or other major organs; and cognitive impairment preventing reliable completion of questionnaire-based assessments.

### Instruments

#### Pediatric rhinoconjunctivitis quality of life questionnaire (PRQLQ)

The PRQLQ is a standardized tool to evaluate quality of life in children with AR. The PRQLQ assesses 23 items across five domains: nose symptoms, eye symptoms, practical problems, other symptoms, and activity limitation. Each item is rated from 0 to 6, with higher scores reflecting greater impairment (total score range, 0–138).

#### Reliability and validity

To verify PRQLQ applicability, 20 children were randomly selected for retesting after a 3-day interval. Test–retest correlation coefficients for the total score and each domain are shown in Table 1. Cronbach's *α* and Guttman split-half reliability coefficients are presented in Table 2. Content validity was assessed by comparing each item's correlation with its hypothesized domain vs. other domains. In this cohort, most items showed moderate-to-high correlations with their hypothesized domains and generally lower correlations with non-hypothesized domains, supporting acceptable item-domain validity (Table 3). The overall test–retest correlation coefficient and Cronbach's α were 0.895 and 0.891, respectively.

**Table 1:** Test-retest reliability of the pediatric rhinoconjunctivitis quality of life questionnaire (PRQLQ) in children with allergic rhinitis.

Reliability Indicators	Overall Dimension	Nose Symptoms	Eye Symptoms	Practical Problems	Other Symptoms	Activity Limitation
Retest correlation coefficient	0.895	0.856	0.886	0.902	0.877	0.888

PRQLQ, pediatric rhinoconjunctivitis quality of life questionnaire.

**Table 2:** Internal consistency and split-half reliability indices of the pediatric rhinoconjunctivitis quality of life questionnaire (PRQLQ).

Reliability Indicators	Total Dimension	Nose Symptoms	Eye Symptoms	Practical Problems	Other Symptoms	Activity Limitation
Cronbach's alpha	0.891	0.872	0.820	0.879	0.935	0.911
Guttman coefficient	0.793	0.734	0.820	0.743	0.823	0.862

PRQLQ, pediatric rhinoconjunctivitis quality of life questionnaire.

**Table 3:** Item-domain correlation matrix for validity evaluation of the pediatric rhinoconjunctivitis quality of life questionnaire (PRQLQ).

Variable	Nose Symptoms	Eye Symptoms	Practical Problems	Other Symptoms	Activity Limitation
Nasal congestion	0.833	0.673	0.371	0.493	0.308
Sneezing	0.851	0.571	0.464	0.364	0.410
Runny nose	0.803	0.544	0.320	0.451	0.383
Nasal itching	0.812	0.651	0.479	0.372	0.457
Eye itching	0.563	0.733	0.441	0.419	0.388
Tearful	0.581	0.811	-	0.328	0.474
Eye swelling	0.584	0.726	-	0.394	0.313
Eye pain	0.471	0.707	0.308	0.483	0.484
Rubbing eyes and nose	0.454	0.703	0.615	0.344	0.398
Blow your nose	0.420	0.385	0.703	0.495	0.365
Carry tissues	0.417	0.651	0.628	0.418	0.403
Taking antihistamines	0.479	0.342	0.751	0.491	0.414
Thirst	0.238	0.380	-	0.707	0.427
Sore throat and hoarseness	0.428	0.374	0.439	0.706	0.338
Headache	0.364	0.373	0.496	0.689	0.510
Fatigue	0.383	0.394	-	0.671	0.369
Feeling unwell all over	0.392	-	0.472	0.637	-
Bad temper	0.374	0.418	0.394	0.583	-
Awkward	0.383	-	0.692	0.507	-
Difficulty concentrating	0.473	0.383	0.414	0.500	0.736
Outdoor activities restricted	0.268	0.368	0.440	0.457	0.769
Difficulty falling asleep	0.460	0.411	0.331	0.439	0.641
Night Awakening	0.487	0.439	0.418	0.314	0.599

Values are item-domain correlation coefficients. A dash indicates that the correlation was not calculated or was not applicable. PRQLQ, pediatric rhinoconjunctivitis quality of life questionnaire.

#### Visual analog scale (VAS)

A 100-mm visual analog scale (VAS), presented as a 10-cm horizontal line, was used to assess symptom severity. Scores ranged from 0 mm (“no symptom”) to 100 mm (“most severe symptom”), with higher scores indicating greater symptom burden. For comparisons of VAS scores across symptoms within the AR group, repeated-measures analysis of variance was used. When overall differences were significant, pairwise comparisons were adjusted using the Bonferroni correction. The full VAS questionnaire is provided in Supplementary Table S1.

#### Questionnaire on factors influencing AR

A structured questionnaire was used to capture potential determinants of AR-related burden, including household environment [pet ownership [yes/no], exposure to tobacco smoke [≥1 cigarette/day indoors], indoor humidity [% measured by hygrometer]], allergen exposure [frequency of outdoor activities during pollen season (times/week), dust-mite exposure intensity, categorized as high or low according to bedding-cleaning frequency and household dust-control practices], and treatment-related factors [medication adherence [percentage of days taken as prescribed in the past month], history of allergen immunotherapy [yes/no]]. The complete questionnaire is provided in Supplementary Table S2.

#### Psychological and sleep assessments

Anxiety and sleep quality were formally assessed using standardized instruments: the Hospital Anxiety and Depression Scale–Anxiety subscale (HADS-A) and the Pittsburgh Sleep Quality Index (PSQI). HADS-A score was entered as a continuous psychosocial variable, whereas poor sleep quality was defined as PSQI > 5 and entered as a binary variable. By contrast, brief anxiety- and sleep-related items in the AR influence questionnaire were used only for descriptive purposes and were not substituted for standardized scores in primary analyses.

#### Survey procedures

Otorhinolaryngology nurses were trained to ensure standardized explanation of questionnaire items. In the clinic, parents/guardians completed the PRQLQ and AR influence questionnaire (∼15 min). Children completed VAS ratings in a quiet room with researcher guidance (∼5 min). All questionnaires were checked the same day for completeness; missing items were clarified immediately. Control-group children were matched for age and sex. Questionnaire completion was supervised to ensure accuracy and consistency.

### Statistical analysis

All analyses were performed using SPSS version 26.0 (IBM Corp., Armonk, NY, USA). Continuous variables are presented as mean ± standard deviation (SD), and categorical variables as frequency (percentage). Between-group comparisons were performed using independent-samples t tests for continuous variables. Comparisons of VAS scores across symptoms within the AR group were performed using repeated-measures analysis of variance, with Bonferroni correction applied for multiple pairwise comparisons.

Multiple linear regression was performed with PRQLQ total score as the dependent variable. Independent variables included household environmental factors, allergen exposure, treatment-related factors, and psychosocial variables. Anxiety was represented by the HADS-A score, whereas sleep quality was represented by poor sleep quality defined as PSQI > 5. Brief background items on emotional status and sleep in the AR questionnaire were used only for descriptive purposes. A stepwise method was employed, with an entry criterion of *P* < 0.05 and a removal criterion of *P* > 0.10, and categorical variables (e.g., pet ownership, allergen immunotherapy history) were dummy-coded (0 = no, 1 = yes).

Multicollinearity was assessed via variance inflation factors (VIF < 5), though we acknowledge that in real-world households, factors such as secondhand smoke and indoor humidity may co-vary (e.g., poorly ventilated homes may have higher humidity and greater smoke exposure). The sample size (*n* = 110) satisfied the approximate requirement of 10–15 subjects per independent variable with fewer than 10 predictors, minimizing overfitting risk. For descriptive item-level analysis, a descriptive item-level impact index was calculated as the proportion of affected children multiplied by the mean score among affected children for each PRQLQ item. This index was used only to rank the relative item-level burden and was not used to calculate PRQLQ total or domain scores.

## Results

### Participant characteristics

A total of 110 children with AR and 110 age- and sex-matched healthy controls were included in the final analysis. The children were aged 6–12 years, with a mean age of 10.22 ± 1.70 years. In the observation group, the duration of AR ranged from 0.5 to 5 years, with a mean disease duration of 2.20 ± 1.10 years. The proportion of boys and girls did not differ significantly between groups (*P* > 0.05).

### Symptom severity based on VAS

Among children with AR, VAS scores for the four core nasal symptoms were similar in magnitude. The mean scores for nasal congestion, nasal itching, sneezing, and rhinorrhea were 54.65 ± 28.36 mm, 52.64 ± 26.79 mm, 52.19 ± 25.92 mm, and 50.18 ± 24.67 mm, respectively (repeated-measures ANOVA: F = 0.53, *P* > 0.05). In contrast, secondary symptoms had significantly lower VAS scores than the core nasal symptoms after Bonferroni correction (F = 205.70, *P* < 0.01; Table 4).

**Table 4:** Visual analog scale (VAS) symptom scores in children with allergic rhinitis, presented as mean ± SD, mm.

Symptom	VAS score (mm)	Symptom	VAS score (mm)
Nasal congestion	54.65 ± 28.36	Eye redness and swelling	5.47 ± 3.42
Nasal itching	52.64 ± 26.79	Eye pain	3.59 ± 2.35
Sneezing	52.19 ± 25.92	Cough	10.17 ± 7.66
Runny nose	50.18 ± 24.67	Shortness of breath	3.14 ± 1.79
Eye itching	33.65 ± 20.69	Wheezing	3.58 ± 1.75
Tearing	10.35 ± 8.44	Pressure sensation	2.01 ± 0.87

Multiple comparisons were adjusted using the Bonferroni correction. Primary nose symptoms showed *P* > 0.05/6 = 0.0083 (not statistically significant), whereas secondary symptoms compared with primary symptoms showed *P* < 0.0083 (statistically significant). SD, standard deviation; VAS, visual analog scale.

### PRQLQ scores and group comparisons

The Chinese version of the PRQLQ showed good reliability in this cohort. The overall test–retest correlation coefficient was 0.895, with domain-specific coefficients ranging from 0.856 to 0.902. The overall Cronbach's α was 0.891, with domain-specific values ranging from 0.820 to 0.935. As shown in Table 5, PRQLQ total scores were significantly higher in children with AR than in healthy controls, indicating poorer HRQoL in the AR group. All five PRQLQ domains—including nose symptoms, eye symptoms, practical problems, activity limitation, and other symptoms—were significantly more impaired in the AR group (all *P* < 0.01). Children with AR most frequently reported nasal obstruction, sneezing, rubbing the nose, and rhinorrhea as the most bothersome problems, whereas eye pain was among the least frequently reported symptoms.

**Table 5:** Comparison of pediatric rhinoconjunctivitis quality of life questionnaire (PRQLQ) scores between children with allergic rhinitis and healthy controls (*n* = 110 per group), presented as mean ± SD.

Group	Total Score	Nose Symptoms	Eye Symptoms	Practical Problems	Other Symptoms	Activity Limitation
Observation Group	53.36 ± 9.42	8.73 ± 1.24	9.51 ± 1.08	12.93 ± 2.78	13.89 ± 3.17	8.30 ± 1.97
Control group	15.94 ± 2.25	3.70 ± 0.85	2.30 ± 0.56	3.45 ± 0.63	3.79 ± 1.11	2.70 ± 0.86
*P*-value	<0.01	<0.01	<0.01	<0.01	<0.01	<0.01

Values are presented as mean ± SD. *P* values were calculated using independent-samples t tests. PRQLQ, pediatric rhinoconjunctivitis quality of life questionnaire; SD, standard deviation.

### Determinants of HRQoL in children with AR

Multiple linear regression analysis was performed to identify factors independently associated with PRQLQ total scores in the AR group (Table 6). Household pet ownership, exposure to secondhand smoke, and high indoor humidity (>60%) were all significantly associated with higher PRQLQ scores, indicating worse HRQoL. More frequent outdoor activities during the pollen season and higher proxy measures of dust-mite exposure were also significant risk factors. Poor medication adherence emerged as one of the strongest predictors of impaired HRQoL, while a history of allergen immunotherapy was associated with lower PRQLQ scores, suggesting a protective effect. In addition, higher anxiety scores and poorer sleep quality were independently related to higher PRQLQ total scores, underscoring the interplay between psychological well-being, sleep, and HRQoL in children with AR. The final regression model explained approximately 62% of the variance in PRQLQ total scores, and all VIF values were below 5, indicating no significant multicollinearity among predictors.

**Table 6:** Multivariable linear regression analysis of factors associated with health-related quality of life (HRQoL) in children with allergic rhinitis (*n* = 110).

Variable Category	Influencing Factor	*β* (Standardized)	*P*-value
Family Environment	Pet ownership (yes vs. no)	0.32	0.004
	Secondhand smoke exposure (Exposed vs. Non-exposed)	0.41	<0.001
	Residential humidity (>60% vs. ≤60%)	0.28	0.001
Allergen exposure	Number of days exposed during pollen season	0.35	0.002
	Dust mite exposure frequency (High vs. Low)	0.39	<0.001
Treatment status	Medication adherence (Poor vs. Good)	0.45	<0.001
	History of immunotherapy (Yes vs. No)	−0.31	0.006
Psychosocial	Anxiety Score (HADS-A)	0.29	<0.001
	Sleep Quality (PSQI > 5 points)	0.48	<0.001

The dependent variable was the PRQLQ total score, with higher scores indicating poorer HRQoL. β denotes the standardized regression coefficient. The model-adjusted R^2^ was 0.62 (F = 18.45, *P* < 0.001), and all VIF values were <5. HADS-A, hospital anxiety and depression scale-anxiety subscale; HRQoL, health-related quality of life; PRQLQ, pediatric rhinoconjunctivitis quality of life questionnaire; PSQI, pittsburgh sleep quality index; VIF, variance inflation factor.

### Distribution of quality of life impacts in the observation group

Analysis of PRQLQ item-level scores showed that nasal congestion affected the highest proportion of children (101/110, 92%) and had the highest descriptive item-level impact index (2.51). Other items with high descriptive item-level impact indices included eye itching (86% affected; index, 2.40), rubbing eyes and nose (81%; 2.20), sneezing (83%; 2.16), blowing one's nose (69%; 2.08), and runny nose (84%; 2.03). The highest mean score among affected children was observed for blowing one's nose (3.01), followed by eye itching (2.79) and nasal congestion (2.73). Lower descriptive item-level impact indices were observed for swollen eyes (46%; 0.88), eye pain (39%; 0.89), and nighttime awakening (60%; 0.91). See Table 7.

**Table 7:** Descriptive item-level impact index for pediatric rhinoconjunctivitis quality of life questionnaire (PRQLQ) items among children with allergic rhinitis.

Variable	Affected children, n	Affected children, proportion	Mean score among affected children	Descriptive item-level impact index
Nasal congestion	101	0.92	2.73	2.51
Sneezing	91	0.83	2.60	2.16
Runny nose	92	0.84	2.42	2.03
Nasal itching	59	0.54	1.80	0.97
Eye itching	95	0.86	2.79	2.40
Teary eyes	63	0.57	1.76	1.00
Swollen eyes	51	0.46	1.91	0.88
Eye pain	43	0.39	2.29	0.89
Rubbing eyes and nose	89	0.81	2.71	2.20
Blowing one's nose	76	0.69	3.01	2.08
Carrying tissues	75	0.68	2.21	1.50
Taking antihistamines	90	0.82	1.31	1.07
Thirst	69	0.63	2.01	1.27
Sore throat and hoarseness	43	0.39	2.49	0.97
Headache	59	0.54	1.71	0.92
Fatigue	68	0.62	1.79	1.11
General discomfort	53	0.48	2.60	1.25
Bad temper	57	0.52	2.12	1.10
Awkward	40	0.36	2.59	0.93
Difficulty concentrating	50	0.45	2.29	1.03
Limited outdoor activities	59	0.54	1.81	0.98
Difficulty falling asleep	56	0.51	2.09	1.07
Nighttime awakening	66	0.60	1.51	0.91

“Affected children” refers to children who reported being affected by the corresponding PRQLQ item. The mean score was calculated only among affected children. The descriptive item-level impact index was calculated as the proportion of affected children multiplied by the mean score among affected children. This index was used only to rank the relative item-level burden and was not intended to be summed or used to calculate PRQLQ total or domain scores. PRQLQ, pediatric rhinoconjunctivitis quality of life questionnaire.

## Discussion

Based on PRQLQ scores, this study demonstrates that AR significantly impairs health-related quality of life (HRQoL) in children aged 6–12 years. PRQLQ total scores and all five domain scores were markedly higher in the observation group than in healthy controls (*P* < 0.01), indicating that AR has a broad impact on both physical and psychosocial functioning. Nose symptoms—particularly nasal obstruction—emerged as the most detrimental component of HRQoL: 92% of children reported being bothered by nasal congestion, with a descriptive item-level impact index as high as 2.51. This pattern is consistent with the known symptom profile of pediatric AR (9). The impact of nasal obstruction on HRQoL appeared greater in magnitude than that of sneezing, which may be related to the relatively narrow nasal cavity and the tendency toward mucosal edema in children, leading to impaired airflow, sleep disruption, and daytime cognitive difficulties (10, 11).

Multivariable regression analysis further identified several key, modifiable determinants of HRQoL in children with AR. Among household environmental factors, pet ownership (β=0.32, *P* = 0.004) was associated with greater impairment, likely due to increased exposure to animal dander and subsequent IgE-mediated inflammatory responses (12). Exposure to secondhand smoke (β=0.41, *P* < 0.001) and living in a high-humidity environment (β=0.28, *P* = 0.001) were also major risk factors; the former can enhance Th2-skewed immune responses, whereas the latter promotes dust-mite proliferation. These findings suggest that preventive strategies should prioritize environmental control, such as avoiding indoor tobacco smoke and maintaining indoor relative humidity below 60%. In terms of allergen exposure, more frequent outdoor activities during the pollen season (β=0.35, *P* = 0.002) and higher dust-mite exposure (β=0.39, *P* < 0.001) were directly related to worse HRQoL, which is consistent with the mechanism whereby ongoing allergen stimulation drives repeated release of inflammatory mediators (3). Intervention strategies may therefore include limiting outdoor activities during peak pollen seasons and changing bedding more frequently to reduce dust-mite exposure.

From a treatment perspective, poor medication adherence (β=0.45, *P* < 0.001) clearly exacerbated disease burden, whereas children who had received allergen immunotherapy had less impairment in HRQoL (β=−0.31, *P* = 0.006). These findings underscore the importance of guideline-based pharmacologic therapy and timely initiation of allergen-specific immunotherapy for improving quality of life (13). Finally, psychosocial factors such as higher anxiety scores on the HADS-A (β = 0.29, *P* < 0.001) and poor sleep quality (PSQI > 5; β = 0.48, *P* < 0.001) were also strongly associated with worse HRQoL. This relationship is likely bidirectional rather than unidirectional. On the one hand, chronic nasal obstruction, repeated sneezing, rhinorrhea, and sleep disruption may directly contribute to emotional distress, irritability, impaired concentration, and social embarrassment in daily life and at school. On the other hand, pre-existing psychological stress may also amplify symptom perception and inflammatory responses through neuro-immune mechanisms, including stress-related autonomic dysregulation and enhancement of inflammatory signaling. This reciprocal interaction may partly explain why anxiety and poor sleep remained independently associated with worse HRQoL even after adjustment for environmental and treatment-related factors. These findings suggest that management of pediatric AR should not be limited to symptom control alone, but should also include attention to emotional well-being, sleep quality, and family-based psychosocial support (5–7). Accordingly, comprehensive management plans should incorporate psychological support and sleep-focused interventions in addition to somatic treatment.

To improve the clinical applicability of these findings, we translated the proposed symptom–environment–behavior framework into a practical clinical pathway that links the major determinants identified in this study to corresponding intervention strategies (Table 8). Taken together, our findings suggest that improving HRQoL in children with AR requires a three-level strategy spanning symptom control, preventive interventions, and integrated management (14). First, dominant symptoms such as nasal obstruction should be treated actively—for example, through the regular use of intranasal corticosteroids and isotonic saline irrigation to relieve congestion and reduce mucosal edema (3, 11). Second, a preventive framework should be established by educating parents to recognize and avoid common environmental triggers, including pet dander, tobacco smoke, and dust mites (12). For children with more severe disease or at higher risk, allergen-specific immunotherapy can be considered to reduce symptom burden and lower the likelihood of progression to asthma (13). Third, cross-dimensional support should be implemented—for instance, encouraging children and families to keep symptom diaries, promoting swimming and other appropriate forms of exercise to improve nasal and respiratory function (15), and providing psychological counseling and practical guidance on improving sleep.

**Table 8:** Clinical pathway linking study-identified determinants to management strategies for health-related quality of life (HRQoL) impairment in children with allergic rhinitis.

Dimension	Key determinant identified in the present study	Clinical implication	Suggested intervention pathway
Symptom burden	Nasal congestion	Major contributor to impaired HRQoL, sleep disruption, and daytime dysfunction	Initiate or optimize intranasal corticosteroid therapy as first-line anti-inflammatory treatment; consider adjunctive isotonic saline irrigation to improve nasal clearance and symptom relief; monitor symptom burden with VAS during follow-up
Household environment	Household pet ownership/pet dander exposure	May increase allergen burden in sensitized children	Assess sensitization history; if clinically relevant, reduce direct exposure to pet dander, strengthen household cleaning measures, and provide family counseling on exposure reduction
Household environment	Secondhand smoke exposure	May aggravate airway inflammation and worsen symptom control	Implement a strict smoke-free home policy; provide caregiver education on tobacco-related symptom aggravation
Household environment	Indoor humidity >60%	Favors dust mite proliferation and persistent indoor allergen exposure	Maintain indoor relative humidity below 60%; improve ventilation and reduce damp indoor conditions
Allergen exposure	Frequent outdoor activities during the pollen season	May increase seasonal allergen exposure and symptom flares	Limit outdoor activities during peak pollen periods when feasible; use exposure-avoidance counseling and reinforce post-exposure hygiene measures
Allergen exposure	High dust mite exposure	Sustains perennial allergen stimulation	Increase the frequency of washing/changing bedding; implement practical mite-reduction measures in the child's bedroom
Treatment-related factors	Poor medication adherence	Leads to suboptimal control despite available therapy	Provide adherence education for children and caregivers; simplify treatment routines when possible; reinforce regular use of prescribed intranasal therapy
Treatment-related factors	No history of allergen immunotherapy	Missed opportunity for disease-modifying treatment in selected patients	Consider allergen immunotherapy in appropriately evaluated children with relevant sensitization and persistent symptoms despite standard management
Psychosocial factors	Elevated anxiety score	May amplify perceived symptom burden and worsen HRQoL	Screen for emotional distress; provide family-based counseling and, when indicated, referral for psychological support
Psychosocial factors	Poor sleep quality	Associated with worse HRQoL and impaired daytime functioning	Optimize nocturnal symptom control, especially nasal obstruction; provide sleep-hygiene guidance and monitor sleep-related complaints during follow-up

This clinical pathway was constructed by integrating the major determinants identified in the present study with current evidence-based management principles for pediatric allergic rhinitis. It is intended to serve as a practical framework rather than a prescriptive treatment algorithm; individual management should still be guided by symptom severity, sensitization profile, comorbidities, and clinical judgment. HRQoL, health-related quality of life; VAS, visual analog scale.

A noteworthy feature of this study is the integration of symptom, environmental, and behavioral dimensions into a single analytical framework, which offers a useful basis for developing precision, multidimensional intervention strategies for pediatric AR. In addition, the mean disease duration of AR in the observation group was 2.20 ± 1.10 years, suggesting that the present findings primarily reflect children with established disease rather than newly diagnosed cases. Nevertheless, as this study was conducted in a single tertiary center, the findings may not fully represent children in other geographic or healthcare settings, and multi-center validation is warranted. Furthermore, the absence of long-term follow-up limits our ability to assess how these HRQoL impairments and determinant effects evolve over time. The cross-sectional design precludes firm conclusions about causality, and the observed associations need to be confirmed in longitudinal studies. In addition, because our sample was drawn from a tertiary hospital, children with milder symptoms or those who did not seek medical care may have been underrepresented. Future research should therefore extend to community-based populations to improve generalizability and further validate these findings.

## Data Availability

The original contributions presented in the study are included in the article/Supplementary Material, further inquiries can be directed to the corresponding author.
