# Supplementary material for: Health-related quality of life and its determinants in children with allergic rhinitis
Source: Front Pediatr. 2026 Jun 3;14:1817538. doi: 10.3389/fped.2026.1817538 (PMC13272351; doi:10.3389/fped.2026.1817538)
Supplement: Supplementary file 1 [file Supplementaryfile1.docx]

Supplementary Material
Supplementary Tables S1. Visual Analog Scale (VAS) Form
Supplementary Tables S2. Questionnaire on Factors Influencing Allergic Rhinitis in Children

*For the manuscript “Health-Related Quality of Life and Its Determinants in Children With Allergic Rhinitis”*

# Supplementary Tables S1. Visual Analog Scale (VAS) Form for Symptoms of Pediatric Allergic Rhinitis

**Target respondent:** Children aged 6–12 years, completed with assistance from research staff and/or a parent or legal guardian when needed.
**Recall period:** Please rate symptoms based on the average severity over the past 7 days.
**Scoring instruction:** Scores ranged from 0 mm (“no symptom”) to 100 mm (“most severe symptom”), with higher scores indicating greater symptom burden.

## Section A. Core Nose Symptoms (primary analysis)

| **Item** | **Symptom** | **VAS (0–100)** |
| --- | --- | --- |
| 1 | Nasal congestion | 0 10 20 30 40 50 60 70 80 90 100 |
| 2 | Rhinorrhea (runny nose) | 0 10 20 30 40 50 60 70 80 90 100 |
| 3 | Sneezing | 0 10 20 30 40 50 60 70 80 90 100 |
| 4 | Nasal itching | 0 10 20 30 40 50 60 70 80 90 100 |

## Section B. Secondary associated symptoms (supplementary analysis)

| **Item** | **Symptom** | **VAS (0–100)** |
| --- | --- | --- |
| 5 | Eye itching | 0 10 20 30 40 50 60 70 80 90 100 |
| 6 | Tearing / watery eyes | 0 10 20 30 40 50 60 70 80 90 100 |
| 7 | Eye redness or swelling | 0 10 20 30 40 50 60 70 80 90 100 |
| 8 | Eye pain or eye discomfort | 0 10 20 30 40 50 60 70 80 90 100 |
| 9 | Cough | 0 10 20 30 40 50 60 70 80 90 100 |
| 10 | Chest tightness / shortness of breath | 0 10 20 30 40 50 60 70 80 90 100 |
| 11 | Wheezing | 0 10 20 30 40 50 60 70 80 90 100 |
| 12 | Facial pressure | 0 10 20 30 40 50 60 70 80 90 100 |

**Note for supplementary use:** In the main analysis of this study, the 4 core Nose Symptoms were treated as primary variables. The secondary symptoms above may be reported descriptively or used for supplementary analyses, depending on the study design.

# Supplementary Tables S2. Questionnaire on Factors Influencing Allergic Rhinitis in Children

**Target respondent:** Parent or legal guardian.
**Estimated completion time:** Approximately 5–8 minutes.
**Instruction:** Please answer the following questions based on the child’s actual situation over the past month, unless otherwise specified.

## Part I. Basic information

Participant ID: ____________________

Sex: □ Male □ Female

Age: ______ years

Duration of allergic rhinitis: ______ months / ______ years

Comorbid allergic disease(s): □ None □ Asthma □ Atopic dermatitis □ Allergic conjunctivitis □ Other: __________

Family history of allergy: □ No □ Yes

## Part II. Household environmental factors

1. Does the family currently keep any pets at home? □ No □ Yes

If yes, specify: □ Cat □ Dog □ Bird □ Other: __________

2. Is the child regularly exposed to pet dander or fur? □ No □ Occasionally □ Frequently

3. Is there any smoker in the household? □ No □ Yes

4. Is the child exposed to secondhand smoke indoors? □ No □ Yes

If yes, estimated exposure level: approximately ______ cigarettes/day

5. Is the home environment humid or damp? □ No □ Yes

6. Indoor relative humidity (if recorded): ______ %

7. Is the home ventilated regularly (e.g., opening windows)? □ Rarely □ Occasionally □ Frequently

8. Are there carpets, plush toys, upholstered fabrics, or similar dust-collecting items in the home? □ No □ Yes

## Part III. Allergen exposure factors

9. During the pollen season, how often does the child participate in outdoor activities per week?

□ 0–1 times □ 2–3 times □ 4–5 times □ ≥6 times

10. During the pollen season, how long is each outdoor activity session on average?

□ <30 minutes □ 30–60 minutes □ 1–2 hours □ >2 hours

11. How often are bedding items (bed sheets, quilt covers, pillow covers) changed?

□ ≥2 times/week □ 1 time/week □ Once every 2 weeks □ Less than once every 2 weeks

12. Are air-conditioner filters cleaned regularly or anti-mite measures taken at home? □ No □ Yes

13. Is the child’s bedroom frequently exposed to dust, old books, blankets, fabric sofas, or similar dust reservoirs? □ No □ Yes

14. Do the child’s symptoms worsen after exposure to a known allergen? □ No □ Yes

If yes, the main suspected allergen(s): ____________________

## Part IV. Treatment-related factors

15. Has the child used any AR-related medication during the past month? □ No □ Yes

16. If yes, which of the following medications or measures were used? (check all that apply)

□ Intranasal corticosteroids □ Oral antihistamines □ Leukotriene receptor antagonists □ Saline nasal irrigation □ Other: __________

17. Over the past month, approximately what proportion of days did the child take medication as prescribed?

□ <25% □ 25%–49% □ 50%–74% □ ≥75%

18. Derived variable for statistical analysis:

Medication adherence = (number of days with correct medication use during the past 30 days / 30) × 100%

19. Has the child ever received allergen immunotherapy (AIT)? □ No □ Yes

20. If yes, what type of immunotherapy was used? □ SCIT □ SLIT □ Not sure

21. If yes, duration of immunotherapy:

□ <6 months □ 6–12 months □ >12 months

## Part V. Psychosocial and sleep background information

**Important note:** The following items are intended for background description only. In formal statistical analyses, anxiety and sleep quality should be quantified using standardized instruments, namely the Hospital Anxiety and Depression Scale–Anxiety subscale (HADS-A) and the Pittsburgh Sleep Quality Index (PSQI), rather than replaced by self-designed items.

22. During the past month, has the child often had difficulty falling asleep because of Nose Symptoms?

□ Never □ Occasionally □ Frequently

23. During the past month, has the child shown irritability, nervousness, or low mood because of Nose Symptoms?

□ Never □ Occasionally □ Frequently

## Suggested statement for the Methods section

The VAS form and the structured questionnaire on factors influencing allergic rhinitis are provided in the Supplementary Material. Anxiety and sleep quality were entered into the formal statistical models using standardized instrument scores (HADS-A and PSQI), while the brief background items in Appendix 2 were used for descriptive support only.
